# Effects of Fasting and Lifestyle Modification in Patients with Metabolic Syndrome: A Randomized Controlled Trial

**DOI:** 10.3390/jcm11164751

**Published:** 2022-08-14

**Authors:** Holger Cramer, Christoph Hohmann, Romy Lauche, Kyung-Eun (Anna) Choi, Nadia Schneider, Nico Steckhan, Florian Rathjens, Dennis Anheyer, Anna Paul, Christel von Scheidt, Thomas Ostermann, Elisabeth Schneider, Daniela A. Koppold-Liebscher, Christian S. Kessler, Gustav Dobos, Andreas Michalsen, Michael Jeitler

**Affiliations:** 1 Department of Internal and Integrative Medicine, Evangelische Kliniken Essen-Mitte, University of Duisburg-Essen, 45276 Essen, Germany; 2 Institute for General Practice and Interprofessional Care, University Hospital Tuebingen, 72076 Tuebingen, Germany; 3 Bosch Health Campus, 70376 Stuttgart, Germany; 4 National Centre for Naturopathic Medicine, Southern Cross University, Lismore 2480, Australia; 5 Institute of Social Medicine, Epidemiology and Health Economics, Charité-Universitätsmedizin Berlin, Corporate Member of Freie Universität Berlin and Humboldt-Universität zu Berlin, 10117 Berlin, Germany; 6 Center for Health Services Research, Brandenburg Medical School Theodor Fontane, 16816 Neuruppin, Germany; 7 Digital Health Center, Hasso Plattner Institute, University of Potsdam, 14469 Potsdam, Germany; 8 Department of Internal and Integrative Medicine, Immanuel Hospital Berlin, 14109 Berlin, Germany; 9 Department of Psychology and Psychotherapy, Witten/Herdecke University, 58455 Witten, Germany

**Keywords:** fasting, metabolic syndrome, modified DASH diet, Mediterranean diet, lifestyle, relaxation, MICOM (mind–body medicine in integrative and complementary medicine)

## Abstract

Background: Lifestyle interventions, such as fasting, diet, and exercise, are increasingly used as a treatment option for patients with metabolic syndrome (MS). This study assesses the efficacy and safety of fasting followed by lifestyle modification in patients with MS compared to lifestyle modification only. Methods: Single-blind, multicenter, parallel, randomized controlled trial in two German tertiary referral hospitals in metropolitan areas. Interventions: (a) 5-day fasting followed by 10 weeks of lifestyle modification (modified DASH diet, exercise, mindfulness; *n* = 73); (b) 10 weeks of lifestyle modification only (*n* = 72). Main outcomes and measures: Co-primary outcomes were ambulatory systolic blood pressure and the homeostasis model assessment (HOMA) index at week 12. Further outcomes included anthropometric, laboratory parameters, and the PROCAM score at weeks 1, 12, and 24. Results: A total of 145 patients with metabolic syndrome (62.8% women; 59.7 ± 9.3 years) were included. No significant group differences occurred for the co-primary outcomes at week 12. However, compared to lifestyle modification only, fasting significantly reduced HOMA index (Δ = −0.8; 95% confidence interval [CI] = −1.7, −0.1), diastolic blood pressure (Δ = −4.8; 95% CI = −5.5, −4.1), BMI (Δ = −1.7; 95% CI = −2.0, −1.4), weight (Δ = −1.7; 95% CI = −2.0, −1.4), waist circumference (Δ = −2.6; 95% CI = −5.0, −0.2), glucose (Δ = −10.3; 95% CI = −19.0, −1.6), insulin (Δ = −2.9; 95% CI = −5.3, −0.4), HbA1c (Δ = −0.2; 95% CI = −0.4, −0.05;), triglycerides (Δ = −48.9; 95% CI = −81.0, −16.9), IL−6 (Δ = −1.2; 95% CI = −2.5, −0.005), and the 10-year risk of acute coronary events (Δ = −4.9; 95% CI = −9.5, −0.4) after week 1. Fasting increased uric acid levels (Δ = 1.0; 95% CI = 0.1, 1.9) and slightly reduced eGRF (Δ = −11.9; 95% CI = −21.8, −2.0). Group differences at week 24 were found for weight (Δ = −2, 7; 95% CI = −4.8, −0.5), BMI (Δ = −1.0; 95% CI = −1.8, −0.3), glucose (Δ = −7.7; 95% CI = −13.5, −1.8), HDL (Δ = 5.1; 95% CI = 1.5, 8.8), and CRP (Δ = 0.2; 95% CI = 0.03, 0.4). No serious adverse events occurred. Conclusions: A beneficial effect at week 24 was found on weight; fasting also induced various positive short-term effects in patients with MS. Fasting can thus be considered a treatment for initializing lifestyle modification for this patient group; however, it remains to be investigated whether and how the multilayered effects of fasting can be maintained in the medium and longer term.

## 1. Introduction

Modern lifestyle leads to an increasing prevalence of type 2 diabetes, metabolic syndrome, and cardiovascular risk constellations [1]. Most risk factors for cardiovascular disease can be influenced by patients’ behavior; this applies above all to poor nutrition, being overweight, lack of exercise, and psychological distress [1]. Epidemiological studies also underline the role of psychological risk factors, such as psychosocial distress, depression, and anxiety, in cardiac health [2,3]. Most coronary patients do not achieve their blood pressure, low-density lipoprotein (LDL) cholesterol, and glucose targets [4]. Moreover, cardiovascular prevention requires advanced preventive cardiological programs delivered by interdisciplinary teams of healthcare professionals, which address all aspects of lifestyle and risk factor management [4].

Lifestyle modification targeting physical inactivity, diet, and psychosocial stress have thus been associated with significant reductions in blood pressure and improvements of other cardiovascular risk factors in risk groups [5,6,7,8,9,10]. There also are hints that combinations of multiple lifestyle modifications might be superior to interventions only targeting a single health behavior [11,12,13]. There is increasing interest and evidence that fasting might substantially reduce cardiovascular risk factors [14,15,16].

There is an increasing number of randomized studies on intermittent fasting in cardiometabolic endpoints, generally lasting 16 to 48 h [16]. Adults who practiced TRE typically lost 1% to 4% of their body weight within several weeks [17,18,19,20]. Furthermore, TRE can improve cardiometabolic endpoints, such as insulin sensitivity and blood pressure [16,21]. Fasting over a longer period, normally from 3 to 21 or more days, has been less studied in humans, although it has a long-standing history in Europe [20]. Fasting is commonly defined as the daily nutritional energy intake of 200 to 500 kcal for up to four weeks [22]. It has been shown to lower blood pressure, blood lipid levels, and other cardiovascular risk factors at least in the short term and appears to be associated with only few adverse events even in diseased populations [22,23]. Animal models of repeated cycles of fasting suggest reductions in mortality and age-associated morbidity, altered signaling, e.g., in signaling pathways of insulin, IGF-1, AMPK, or mTor, as well as enhanced autophagy and ketone body production [24]. Most of these effects have been confirmed in the first human studies on prolonged, periodic, and intermittent fasting as well as fasting-mimicking diets in the meantime [16,24,25,26].

This study aimed to assess the effects of fasting in patients with metabolic syndrome, followed by a multimodal lifestyle modification intervention named MICOM (mind–body medicine in integrative and complementary medicine), compared to lifestyle modification intervention only. We hypothesized a priori that a 5-day fast followed by 10 weeks of lifestyle modification would be more effective for reducing ambulatory systolic blood pressure and the homeostasis model assessment (HOMA) index than lifestyle modification alone.

## 2. Materials and Methods

### 2.1. Design

This single-blind, multicenter, parallel, randomized controlled trial was conducted at the Department of Internal and Integrative Medicine, Evang. Kliniken Essen-Mitte, Essen, Germany and the Department of Internal and Integrative Medicine, Immanuel Hospital Berlin and Charité Outpatient Center for Complementary and Integrative Medicine, Berlin, Germany. The study had been approved by the ethics committees of the Charité-Universitätsmedizin Berlin (approval number: EA4/141/13) and the University of Duisburg-Essen (approval number: 14-5733 BO) and registered at ClinicalTrials.gov (registration number: NCT02099968) prior to patient recruitment. The study was conducted and reported in accordance with the CONSORT (CONsolidated Standards of Reporting Trials) 2010 guidelines [27]. No important changes were made to the study protocol after trial commencement.

### 2.2. Participants

Patients were recruited from study centers and through local newspaper announcements, screened by a research assistant to assess eligibility, and, if apparently eligible, assessed by a study physician. If patients met all inclusion criteria and did not meet any exclusion criteria, informed consent was obtained, and they were included in the trial.

Patients (male/female) with a metabolic syndrome according to the National Expert Panel on Detection, Evaluation, and Treatment of High Blood Cholesterol in Adults (NCEP-ATP-III) criteria were included. Patients were further required to be diagnosed with systolic hypertension and/or with an additional subclinical atherosclerosis (<50% coronary artery stenosis, <50% carotid artery stenosis, or peripheral artery disease stage 1).

Exclusion criteria included: (i) diabetes mellitus type 1 or insulin bolus therapy (c-peptide < 1.2 ng/mL), (ii) coronary artery disease, myocardial infarction, pulmonary embolism, or stroke within the past 3 months, (iii) heart failure ≥ stage I NYHA, (iv) peripheral artery disease ≥ stage 2, (v) chronic kidney disease > stage 2 (GFR < 60 mL/min), (vi) eating disorder, dementia, or psychosis, or (vii) other severe internal diseases.

### 2.3. Interventions

Both interventions were group-based and conducted by a multidisciplinary team of healthcare professionals (nutritional counselors, mind–body therapists, or sport therapists) with board certified education. They incorporated aspects of the mind–body program, which was originally developed by the Benson-Henry Mind/Body Medical Institute at Harvard Medical School and further developed and adapted to the German needs, as described in the MICOM (mind–body medicine in integrative and complementary medicine) program [28]. This program focuses on mindfulness and specific group training that are rooted in psychoneuroendocrinology and use formal meditation and gentle yoga exercises. Nutritional education included counseling, comprehensive lectures, and cooking classes.

#### 2.3.1. Fasting and Lifestyle Modification (F + LM)

Patients in this group started with two calorie-restricted vegan days (max 1200 kcal/day), followed by a 5-day modified fasting intervention (intake of 300–350 kcal/day, obtained from vegetable juices and vegetable broth). Thereafter, a stepwise reintroduction of food according to published guidelines of fasting was performed, followed by a group dietary and lifestyle modification intervention [29] (weekly 6-h multimodal sessions for a total of 10 weeks). This included lectures and teaching kitchens on a vegetarian whole-food diet with a focus on a plant-based Mediterranean diet and a modified DASH diet [30,31,32], intermittent fasting (rice only days once/week) [33], and recommendations for specific cardioprotective foods, such as beetroot, nuts, and olive oil.

Each session started with 20–30 min of activating exercises, and moderate aerobic exercise, i.e., walking, was introduced in a 45-min supervised training at one of the first sessions. Each session closed with a supervised training in stress reduction using progressive muscle relaxation, mindfulness meditation, or yoga. Mindfulness was further incorporated in a 90-min supervised training at one of the first sessions. The mindfulness session included a theoretical introduction into the concept of mindfulness combined with practical exercises of meditation, yoga, qigong, body scan, etc. Mindfulness in everyday life was specifically targeted during homework, where participants are taught to be mindful during routine activities, such as eating, talking, or working.

Home practice outside the session was encouraged for both aerobic exercise and relaxation/mindfulness training.

After the end of the program, in weeks 13 to 24, monthly group sessions were offered to ensure adherence.

#### 2.3.2. Lifestyle Modification (LM)

The lifestyle intervention was similar to the one performed for the fasting and lifestyle modification group, without the fasting intervention. The program consisted of 55 h of group intervention over a period of 10 weeks. After the end of the program, in months 13 to 24, monthly group sessions were offered to ensure adherence.

### 2.4. Randomization

Patients were randomly allocated 1:1 to F + LM or LM by block-randomization with randomly varying block lengths, stratified by (a) study center and (b) the intake/nonintake of antihypertensive medication. The randomization list was created by a biometrician not involved in patient recruitment or assessment, using the Random Allocation Software [34]. The list was password-secured, and no person other than the biometrician was able to access it. Based on these results, the sealed, sequentially numbered opaque envelopes containing the treatment assignments were prepared. After obtaining written informed consent and baseline assessment, the study physician opened the lowest numbered envelope to reveal that patient’s assignment.

### 2.5. Outcome Measures

Outcomes were assessed at baseline and at 1, 12, and 24 weeks after randomization by a blinded outcome assessor who was not involved in patient recruitment, allocation, or treatment. Two primary outcome measures were defined: ambulatory systolic blood pressure and the homeostasis model assessment (HOMA) index at week 12. Herein, primary outcomes and further clinical parameters are reported. Further explorative experimental variables (immune function, microbiome) are reported elsewhere [35]. Further psychometric parameters will be reported elsewhere.

#### 2.5.1. Physician-Assessed Outcomes

Twenty-four-hour ambulatory systolic and diastolic blood pressure were measured using an validated digital blood pressure monitor (Mobil-O-Graph^®^ PWA, I.E.M., Stolberg, Germany) [36]. Measurements at week 0 were made within a week before the start, those at week 12 within a week after the end of the intervention at the same time of day at each of the three measurement time points. The monitoring software automatically removed incorrect measurements using built-in algorithms. Clinical blood pressure was measured in the hospital by a sphygmomanometer, using the average of three consecutive measurements. Clinical blood pressure was measured at each time point and ambulatory blood pressure only at baseline at weeks 12 and 24.

Body weight was measured using the Omron BF 511 bioelectrical impedance device [37]. BMI was calculated as the weight in kilograms divided by the square of height in meters. Waist circumference was measured by two research assistants using a measuring tape in the horizontal plane exactly midway between the iliac crest and the costal arch. Measures were repeated twice, and the mean of both measures was used; if the two measures differed by more than 1 cm, both measurements were repeated. Hip circumference was measured in the horizontal plain at the maximal circumference of the hips or buttock region above the gluteal fold, whichever is larger, using the same approach as for waist circumference. Waist–hip ratio was calculated as the quotient of waist circumference and hip circumference [38]. Body fat percentage and muscle mass percentage were measured by bioelectrical impedance analysis using the Omron BF 511 bioelectrical impedance device [37].

#### 2.5.2. Laboratory Measures

Blood samples were collected from the antecubital vein into vacutainer tubes and analyzed using the Modular P analyzer (Roche, Mannheim, Germany). Metabolic parameters included blood glucose levels, blood insulin levels, and HbA1c, and were analyzed using standard procedures. HOMA index was calculated as blood insulin level (µU/mL) × blood glucose level (mmol/L)/22.5 [39]. Further laboratory parameters included blood lipid levels (total cholesterol, HDL cholesterol, LDL cholesterol, and triglyceride), uric acid, blood creatinine level, estimated glomerular filtration rate (eGFR), C-reactive protein (CRP), insulin-like growth factor 1 (IGF-1), and interleukin 6 (IL-6).

#### 2.5.3. PROCAM Score

Cardiovascular risk was calculated by the PROCAM (Prospective Cardiovascular Münster Study) score considering clinical (age, smoking status, diagnosis of diabetes mellitus type 2, systolic blood pressure, intake of antihypertensive medication, myocardial infarction, and/or stroke within the close family) and laboratory parameters (HDL, LDL, triglyceride, and fasting glucose level) [40]. Based on this score, the 10-year risk of an acute coronary event was calculated [40].

### 2.6. Safety

All adverse events occurring during the study period were recorded. Patients experiencing such adverse events were asked to see the study physician to assess their import and initiate any necessary response. Open-ended questions were used at week 1, 12, and week 24 to assess any adverse events not previously mentioned by the patients. Patients were asked to indicate any adverse events during the study period regardless of their severity or potential relationship to the study intervention.

### 2.7. Sample Size Calculation and Statistical Analysis

The required sample size was calculated a priori using G*Power software [41]. Based on prior research on multimodal lifestyle interventions [6,7], such as yoga [42], mindfulness [43], and Mediterranean diet [44], a between-group effect size of d = 0.5 was expected. A level 2.5% *t*-test requires a total of 64 patients per group to detect a respective group difference with a statistical power of 80%. Accounting for a potential loss in power because of a maximum of 10% dropouts, it was planned to include at least 142 patients in this trial.

All analyses were based on an intention-to-treat basis, including all participants being randomized, regardless of whether they provided a full set of data or adhered to the study protocol. Missing data were imputed by multiple means using Markov chain Monte Carlo methods [45,46], yielding a total of 50 complete datasets.

Group differences were analyzed by univariate analyses of covariance (ANCOVA), which modeled the outcome at week 1, 12, or 24 as a function of the treatment group (classified factor), the stratification factors study center (classified covariate), baseline antihypertensive medication intake (classified covariate), and the respective baseline value (linear covariate). Afterward, the 50 estimates of group differences were combined to produce overall effect size estimates, 95% confidence intervals, and *p*-values.

Thus, the analysis accounted for potential baseline differences in medication and in the respective outcomes. Inferential statistical tests for baseline differences between groups were not conducted because the CONSORT statement explicitly discourages such tests, given that baseline differences in a randomized trial are generally considered to be random.

For the primary and secondary outcomes, *p*-values ≤ 0.025 and ≤0.050, respectively, were considered significant.

All analyses were performed using the Statistical Package for Social Sciences software (IBM SPSS Statistics for Windows, release 22.0, IBM Group, Armonk, NY, USA).

## 3. Results

### 3.1. Patients

**Figure 1:**
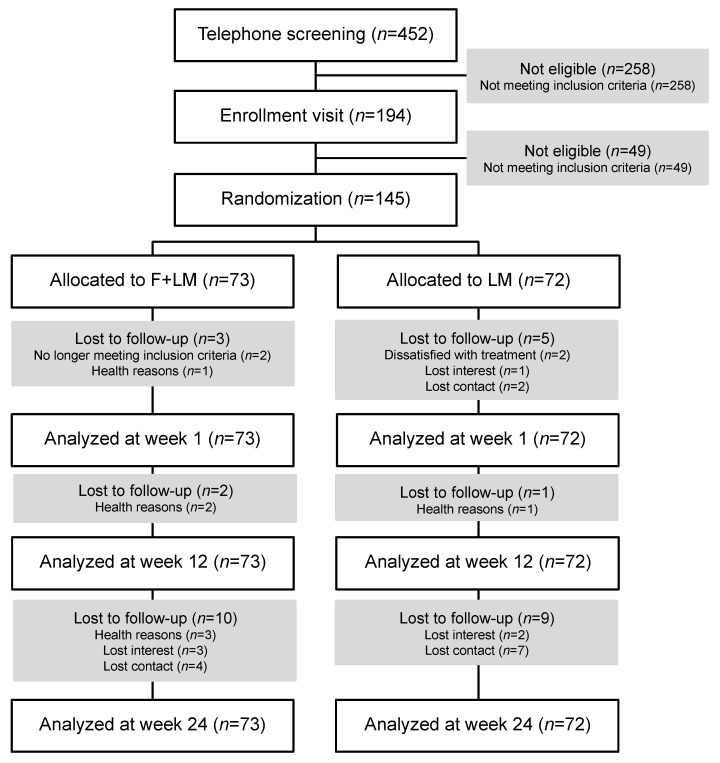
Study flow chart. Abbreviations: F + LM, fasting and lifestyle modification; LM, lifestyle modification.

A total of 452 patients were telephone screened, and 258 were excluded because they did not meet the inclusion criteria (Figure 1). A total of 49 patients were excluded after medical assessment. A total of 145 patients were enrolled after providing informed consent and were randomized to the F + LM group (*n* = 73) or the LM group (*n* = 72). During the study period, 15 patients each were omitted during follow-up in the F + LM and LM groups (Figure 1). Participants’ mean age was 59.7 ± 9.3 in the whole study population (Table 1) and about two-thirds of the participants were female, one-third of those having a university degree. Baseline characteristics were balanced between groups. Patients had a BMI of 33.3 ± 4.5 kg/m^2^ (Table 1). Patients in the F + LM group attended a mean of 8.2 ± 2.3 (82.0%) out of 10 possible intervention sessions; patients in the LM group attended 7.1 ± 3.5 (71%) sessions (*p* = 0.124).

### 3.2. Primary Outcome Measures

The two primary outcome measures, 24-h ambulatory systolic blood pressure and the HOMA index at week 12, showed reductions in both groups and were not significantly different between the groups (Table 2 and Table 3).

### 3.3. Physician-Assessed Outcomes and PROCAM Score

**Figure 2:**
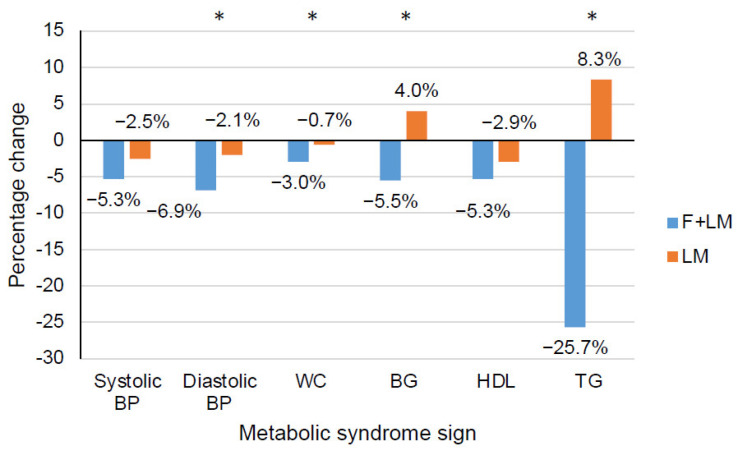
Percentage change in diagnostic signs of the metabolic syndrome after the 5-day fasting period (F + LM) or 5-day waiting period (LM). Abbreviations: BG, blood glucose level; BP, blood pressure; F + LM, fasting and lifestyle modification; HDL, high-density lipoprotein cholesterol; LM, lifestyle modification; TG, triglycerides; WC, waist circumference; *, *p* < 0.05.

At week 1, after the fasting week, the clinical diastolic blood pressure, weight, BMI, waist circumference, and the 10-year risk of acute coronary events (based on the PROCAM score) were significantly lower in the F + LM group than in the LM group (Table 2, Figure 2). While these effects were maintained at week 12, along with further effects on body fat percentage and the PROCAM score, only weight and BMI were lower in the F + LM group at week 24 (Table 2).

### 3.4. Laboratory Parameters

Group differences favoring the F + LM group over the LM group were found after the fasting week for HOMA index, blood glucose level, blood insulin level, HbA1c, triglyceride levels, and IL-6 (Table 3, Figure 2). Uric acid and eGFR significantly worsened in the F + LM group compared to the LM group after the fasting week. None of these group differences persisted at week 12. However, further positive effects favoring F + LM over LM at week 24 were found for blood glucose level and HDL cholesterol (Table 2). CRP was higher in F + LM compared to LM at week 24.

### 3.5. Safety

One serious adverse event each occurred in both groups. In the F + LM group, an acute diverticulitis occurred in a patient with a known diverticulosis; a causal relationship to the study intervention was rated as unlikely. The patient completely recovered after sigmoid resection and stationary treatment. In the LM group, a patient had suffered a “trauma” (physical and psychological), resulting in a hospitalization; further details could not be elicited as the patient did not want to give further information. A total of 73 minor adverse events occurred in 43 patients in the F + LM group, and a total of 51 minor adverse events occurred in 32 patients in the LM group (Table S1 in the Supplementary Material).

## 4. Discussion

No significant between-group differences were found in the two primary outcomes, 24-h systolic blood pressure and HOMA index, at week 12 in patients with metabolic syndrome. However, there were interesting exploratory findings suggesting benefits from fasting in this patient population. In patients initially fasting for 5 days, lower body weight and BMI values were found at all three assessment points compared to the non-fasting group along with further, mainly short-term, anthropometric and laboratory differences. Markedly, all parameters of the metabolic syndrome except for HDL cholesterol showed significant improvement after the fasting period compared to the non-fasting period. Both interventions were safe.

Trial participants were demographically heterogeneous. More than 60% were women. The distributions of educational attainment were broad but slightly skewed toward persons with higher education. The inclusion criteria of the study included a large proportion of patients who were German adults with metabolic syndrome. These aspects of the study suggest that the study results should be applicable to a large proportion of the German population.

The beneficial augmenting cardiometabolic effects of fasting are in line with previous studies [22,23,26]. Calorie restriction and fasting have been argued to reduce cardiovascular risk; however, this was predominantly demonstrated in animal models [47,48], uncontrolled studies [23], and studies including time-restricted eating, intermittent fasting, and fasting-mimicking diets [26]. In a large observational study [23], weight loss increased and abdominal circumference decreased with the length of the fasting period. Beneficial effects on blood pressure, blood lipids, and glycaemia were also shown.

At least in the short-term, the 6% absolute risk reduction in cardiovascular events in this study corroborates these findings. Proposed mechanisms for risk reduction include reductions in age-associated changes in the heart and vessels as well as reduced levels of apoptosis, insulin and IGF-1 signaling, enhanced autophagy, and ischemic preconditioning [24,47].

Minor reduction in calculated glomerular filtration was not persistent and might be attributed to mild protein catabolism [23]. Likewise, increased uric acid levels during fasting are known in the literature but have not been associated with symptomatic gout [22,23]. On the one hand, the increased concentration of uric acid is probably due to a slight initial increase in protein catabolism. On the other hand, once the levels of ketone bodies rise in the serum, uric acid is being retained in the kidneys due to both substances competing for tubular secretion and ketone bodies being preferentially secreted during fasting [49]. Prior studies found no deterioration of renal function [23]. Nevertheless, the effect of fasting on kidney function must be further investigated.

Our study has some important limitations. First, the main limitation is the lack of effects at week 24. Since the primary assessment time point was the end of the lifestyle modification programs, the short-term effects of fasting can be regarded as preliminary only. While the interventions included dietary advice, fasting was used only once and no approaches to maintain the effects of fasting in the medium and longer term, such as regular intermittent fasting or repeated cycles of fasting or fasting-mimicking diets, as recently suggested, were used [15,26]. It seems likely that the mean difference between groups expected in the sample size calculation was chosen too optimistically in view of the relatively strong control group. Accordingly, the lack of effects at week 24 could also be attributed to a lack of power. Second, adherence to the (dietary) interventions was not assessed in detail (e.g., via nutritional protocols). Third, the time intensity of the two programs was slightly different, as more elements were implemented in the F + LM group due to the fasting component.

Against the background of the above-mentioned limitations, future studies should undertake efforts to maintain the fasting effect by repeating fasting cycles or the additive use of intermittent fasting. Hereby, relevant and delineated mechanisms behind the metabolic switch of fasting, such as endocrine and neurobiological effects, autophagy and microbiome-related effects should be considered and translated to clinical protocols [47,50]. Future studies will likely require substantially larger sample sizes to detect group differences in e.g., coronary risk scores, if such effects do exist.

Most health insurance companies do not cover multimodal lifestyle modification interventions for the prevention and treatment of metabolic syndrome. Given the substantial health benefits of lifestyle modification interventions to improve cardiovascular parameters, it is time to consider how such programs might be implemented, particularly for patients at increased cardiovascular risk. The costs of such programs should be weighed against the benefits of preventing heart disease, hypertension, diabetes, and other conditions, thereby avoiding the need for potentially costly medical treatments.

## 5. Conclusions

While the beneficial effects of fasting were not preserved in week 24, fasting induced relevant short-term effects in patients with metabolic syndrome. Fasting can thus be considered as a starting point for treating metabolic syndrome. However, it remains to be investigated how the effects of fasting can be maintained in the medium and longer term. Ultimately, a population-wide adoption of a healthy lifestyle as implemented in the study interventions may reduce the societal burden of cardiovascular diseases.

## Figures and Tables

**Table 1:** Baseline sociodemographic and clinical characteristics. If not otherwise denoted, values are reported as mean ± standard deviation. Abbreviations: F + LM, fasting and lifestyle modification; LM, lifestyle modification.

	Total (*n* = 145)	F + LM (*n* = 73)	LM (*n* = 72)
Sociodemographic characteristics			
Gender female *n* (%)	91 (62.8%)	48 (65.8%)	43 (59.7%)
Age years	59.7 ± 9.3	58.6 ± 10.8	60.8 ± 7.5
Marital status *n* (%)			
Single	15 (10.3%)	6 (8.2%)	9 (12.5%)
Married	98 (67.6%)	49 (67.1%)	49 (68.1%)
Divorced	21 (14.5%)	12 (16.4%)	9 (12.5%)
Widowed	5 (3.4%)	3 (4.1%)	2 (2.8%)
Education *n* (%)			
Secondary modern school (“Hauptschule”) qualification	25 (17.2%)	9 (12.3%)	16 (22.2%)
High school (“Realschule”) qualification	41 (28.3%)	19 (26.0%)	22 (30.6%)
A level (“Abitur”)	18 (12.4%)	12 (16.4%)	6 (8.3%)
University degree	53 (36.6%)	28 (38.3%)	25 (34.7%)
Employment *n* (%)			
Employed full-time	41 (28.3%)	20 (27.4%)	21 (29.2%)
Employed part-time	19 (13.1%)	11 (15.1%)	8 (11.1%)
Occasionally	5 (3.4%)	3 (4.1%)	2 (2.8%)
On sick leave	3 (2.1%)	2 (2.7%)	1 (1.4%)
Unemployed	3 (2.1%)	2 (2.7%)	1 (1.4%)
Retired age-related	44 (30.3%)	20 (27.4%)	24 (33.3%)
Retired health-related	15 (10.3%)	7 (9.6%)	8 (11.1%)
Homekeeper	10 (6.9%)	6 (8.2%)	4 (5.6%)
Clinical characteristics			
Weight kg	97.0 ± 15.8	98.1 ± 16.1	95.9 ± 15.5
Body Mass Index kg/m^2^	33.3 ± 4.5	33.7 ± 4.5	32.84.5
Diagnosis of obesity since months	65.0 ± 123.6	73.3 ± 140.7	56.5 ± 103.8
Diagnosis of hypertension since months	119.5 ± 112.4	119.3 ± 113.8	119.7 ± 111.8
Antihypertensive drugs since months	106.0 ± 108.6	98.7 ± 101.7	113.4 ± 115.4
Diagnosis of coronary artery disease *n* (%)	5 (3.4%)	2 (2.7%)	3 (4.2%)
Diagnosis of peripheral artery disease *n* (%)	0 (0.0%)	0 (0.0%)	0 (0.0%)
Diagnosis of hyperlipidemia since months	62.2 ± 83.1	63.7 ± 91.9	60.7 ± 73.9
Lipid-lowering drugs since months	30.4 ± 59.0	28.3 ± 56.9	32.6 ± 61.4
Diagnosis of hyperglycemia since months	11.5 ± 39.0	10.2 ± 32.6	12.7 ± 44.8
Anti-hyperglycemic drugs since months	17.2 ± 36.1	9.0 ± 24.8	25.6 ± 43.3
Bariatric surgery *n* (%)	0 (0.0%)	0 (0.0%)	0 (0.0%)

**Table 2:** Effects of the study interventions on physician-assessed outcomes, the PROCAM score, and the 10-year risk of acute coronary events based on the PROCAM score. Values are expressed as mean ± standard deviation. Bold *p*-values indicate significant group differences (*p* < 0.05). Abbreviations: BP, blood pressure; CI, confidence interval; F + LM, fasting and lifestyle modification; LM, lifestyle modification; NA, not assessed.

Outcome	Group	Week 0	Week 1	Week 1		Week 12	Week 12		Week 24	Week 24	
				Group Difference (95% CI)	*p*		Group Difference (95% CI)	*p*		Group Difference (95% CI)	*p*
Ambulatory systolic BP	F + LM	131.1 ± 9.1	NA	NA	NA	126.9 ± 8.9	−0.5 (−5.0, 3.9)	0.813	130.0 ± 9.0	−1.2 (−5.6, 2.1)	0.366
	LM	132.5 ± 11.0	NA			129.3 ± 10.8			130.7 ± 9.7		
Ambulatory diastolic BP	F + LM	80.1 ± 8.2	NA	NA	NA	78.4 ± 8.2	−1.3 (−4.8, 2.1)	0.450	79.1 ± 7.6	−1.0 (−4.2, 2.3)	0.501
	LM	82.0 ± 8.3	NA			80.2 ± 8.7			80.6 ± 8.5		
Clinical systolic BP	F + LM	138.9 ± 14.4	130.9 ± 16.1	−4.1 (−11.3, 3.2)	0.270	133.7 ± 13.5	1.6 (−4.4, 7.5)	0.609	138.3 ± 14.4	2.5 (−3.6, 8.5)	0.417
	LM	141.2 ± 19.0	136.2 ± 14.5			134.5 ± 12.3			135.2 ± 10.8		
Clinical diastolic BP	F + LM	88.3 ± 10.6	81.5 ± 9.7	−4.8 (−9.6, −0.06)	0.047	86.5 ± 11.2	3.4 (−0.7, 7.5)	0.106	88.7 ± 10.3	−2.7 (−6.8, 1.4)	0.200
	LM	89.5 ± 11.2	87.0 ± 11.1			86.7 ± 8.7			87.7 ± 8.8		
Weight	F + LM	98.1 ± 16.1	93.2 ± 15.2	−4.8 (−5.5, −4.1)	<0.001	92.8 ± 15.4	−3.5 (−5.1, −1.8)	<0.001	93.3 ± 15.5	−2.7 (−4.8, −0.5)	0.014
	LM	95.9 ± 15.5	95.8 ± 15.3			94.3 ± 15.1			93.8 ± 15.1		
Body Mass Index	F + LM	33.7 ± 4.5	32.1 ± 4.3	−1.7 (−2.0, −1.4)	<0.001	31.9 ± 4.3	−1.3 (−1.9, −0.7)	<0.001	32.1 ± 4.4	−1.0 (−1.8, −0.3)	0.007
	LM	32.8 ± 4.5	32.8 ± 4.4			32.3 ± 4.4			32.1 ± 4.3		
Waist circumference	F + LM	114.1 ± 10.5	110.7 ± 11.2	−2.6 (−5.0, −0.2)	0.035	108.2 ± 11.5	−3.5 (−5.8, −1.1)	0.004	108.9 ± 10.9	−1.2 (−6.3, 3.9)	0.633
	LM	112.1 ± 11.1	111.3 ± 11.1			109.4 ± 10.9			107.2 ± 16.5		
Waist/hip Ratio	F + LM	1.0 ± 0.5	1.0 ± 0.1	−0.01 (−0.05, 0.03)	0.486	1.0 ± 0.1	−0.03 (−0.06, 0.01)	0.192	1.0 ± 0.1	−0.01 (−0.06, 9.04)	0.658
	LM	1.0 ± 0.2	1.0 ± 0.1			1.0 ± 0.1			1.0 ± 0.1		
Body fat percentage	F + LM	41.5 ± 8.9	42.0 ± 9.1	0.6 (−0.8, 1.9)	0.420	39.4 ± 8.9	−2.2 (−3.4, 0.9)	0.001	40.1 ± 9.2	−1.0 (−2.6, 0.6)	0.214
	LM	39.4 ± 8.6	39.9 ± 8.6			39.2 ± 8.7			39.1 ± 8.8		
PROCAM Score	F + LM	48.4 ± 13.6	45.4 ± 13.2	−2.4 (−5.6, 0.8)	0.139	45.8 ± 13.4	−3.4 (−6.7, −0.2)	0.048	47.3 ± 12.7	−2.0 (−5.5, 1.4)	0.242
	LM	50.1 ± 11.8	50.9 ± 13.2			49.7 ± 12.9			50.0 ± 11.6		
10-year coronary risk	F + LM	14.9 ± 12.6	12.1 ± 11.2	−4.9 (−9.5, −0.4)	0.033	12.6 ± 11.8	−6.2 (−10.3, −2.0)	0.004	13.7 ± 12.0	−3.8 (−8.2, 0.5)	0.080
	LM	16.0 ± 13.6	17.4 ± 15.2			15.5 ± 12.9			15.8 ± 12.5		

**Table 3:** Effects of the study interventions on laboratory parameters. Values are expressed as mean ± standard deviation. Bold *p*-values indicate significant group differences (*p* < 0.05). Abbreviations: BP, blood pressure; CI, confidence interval; CRP, C-reactive Protein; eGFR, estimated glomerular filtration rate; F + LM, fasting and lifestyle modification; HbA1c, glycated hemoglobin; HOMA, homeostasis model assessment; IGF, insulin-like growth factors; IL, interleukin; LM, lifestyle modification.

Outcome	Group	Week 0	Week 1	Week 1		Week 12	Week 12		Week 24	Week 24	
				Group Difference (95% CI)	*p*		Group Difference (95% CI)	*p*		Group Difference (95% CI)	*p*
HOMA index	F + LM	3.5 ± 2.5	2.0 ± 1.6	−0.8 (−1.7, −0.1)	0.046	3.2 ± 2.2	0.2 (−0.7, 1.1)	0.676	3.0 ± 1.9	−0.2 (−0.9, 0.6)	0.639
	LM	3.7 ± 2.4	3.4 ± 2.3			3.4 ± 2.2			3.6 ± 2.0		
Blood glucose	F + LM	113.3 ± 18.9	107.0 ± 18.3	−10.3 (−19.0, −1.6)	0.022	106.2 ± 13.4	−7.7 (−17.2, 1.9)	0.113	110.5 ± 12.8	−7.7 (−13.5, −1.8)	0.011
	LM	110.1 ± 22.0	114.4 ± 26.9			109.3 ± 24.4			111.5 ± 20.3		
Blood insulin	F + LM	12.4 ± 7.3	8.0 ± 5.4	−2.9 (−5.3, −0.4)	0.024	11.6 ± 6.6	0.9 (−1.4, 3.3)	0.428	10.9 ± 5.9	−0.5 (−2.7, 1.7)	0.641
	LM	13.0 ± 7.5	12.4 ± 7.0			11.9 ± 5.9			12.4 ± 5.7		
HbA1c	F + LM	5.9 ± 0.5	5.8 ± 0.5	−0.2 (−0.4, −0.05)	0.010	5.8 ± 0.5	−0.08 (−0.3, 0.1)	0.485	5.9 ± 0.4	−0.2 (−0.4, 0.04)	0.122
	LM	5.9 ± 0.7	6.0 ± 0.7			5.9 ± 0.7			6.0 ± 0.7		
Total cholesterol	F + LM	224.4 ± 50.0	208.4 ± 47.6	−6.9 (−25.3, 11.5)	0.458	214.3 ± 40.6	−4.0 (−19.7, 11.7)	0.616	227.7 ± 39.9	9.5 (−9.7, 27.9)	0.339
	LM	224.3 ± 48.3	224.9 ± 47.5			212.8 ± 42.2			216.8 ± 42.0		
HDL cholesterol	F + LM	53.4 ± 16.0	49.2 ± 12.0	−1.1 (−4.4, 2.3)	0.531	53.4 ± 14.7	3.3 (−1.0, 7.7)	0.134	55.3 ± 13.3	5.1 (1.5, 8.8)	0.007
	LM	56.6 ± 19.0	54.1 ± 16.0			53.1 ± 15.7			53.5 ± 15.1		
LDL cholesterol	F + LM	140.2 ± 37.3	134.1 ± 40.3	−0.9 (−16.3, 14.5)	0.904	135.2 ± 34.5	−3.6 (−17.1, 9.9)	0.598	144.9 ± 32.1	7.1 (−7.7, 21.9)	0.344
	LM	139.6 ± 43.5	142.3 ± 42.6			132.8 ± 37.4			137.8 ± 37.5		
Triglyceride	F + LM	188.0 ± 210.6	116.4 ± 53.9	−48.9 (−81.0, −16.9)	0.003	157.4 ± 89.5	−23.0 (−58.2, 12.1)	0.197	157.3 ± 93.9	−21.9 (−59.4, 15.6)	0.250
	LM	175.5 ± 111.1	169.9 ± 93.4			175.3 ± 101.9			161.0 ± 78.4		
Uric acid	F + LM	6.3 ± 1.7	8.0 ± 2.2	1.0 (0.1, 1.9)	0.026	6.3 ± 1.6	−0.1 (−0.9, 0.6)	0.710	6.2 ± 1.6	0.1 (−0.5, 0.7)	0.650
	LM	6.6 ± 1.5	6.7 ± 1.7			6.2 ± 1.5			6.2 ± 1.2		
Creatinine	F + LM	0.9 ± 0.2	1.0 ± 0.2	0.04 (−0.05, 0.1)	0.383	0.9 ± 0.2	−0.03 (−0.1, 0.04)	0.354	0.8 ± 0.2	−0.04 (−0.1, 0.02)	0.187
	LM	0.9 ± 0.2	0.9 ± 0.2			0.9 ± 0.2			0.9 ± 0.2		
eGFR	F + LM	83.5 ± 15.7	73.4 ± 17.5	−11.9 (−21.8, −2.0)	0.019	85.8 ± 13.6	1.4 (−6.4, 9.1)	0.728	86.6 ± 14.6	2.4 (−6.1, 11.0)	0.577
	LM	82.4 ± 14.5	81.5 ± 14.5			82.9 ± 13.6			82.1 ± 12.2		
CRP	F + LM	0.4 ± 0.4	0.5 ± 0.4	0.03 (−0.1, 0.2)	0.677	0.4 ± 0.5	0.04 (−0.1, 0.2)	0.628	0.4 ± 0.5	0.2 (0.03, 0.4)	0.024
	LM	0.3 ± 0.3	0.4 ± 0.3			0.3 ± 0.3			0.4 ± 0.3		
IGF-1	F + LM	119.9 ± 38.1	104.1 ± 40.6	−13.6 (−28.8, 1.5)	0.077	123.7 ± 38.6	−7.6 (−20.2, 5.0)	0.235	126.5 ± 40.6	2.4 (−11.7, 16.6)	0.736
	LM	126.2 ± 48.2	120.0 ± 44.2			129.9 ± 43.7			128.4 ± 43.7		
IL-6	F + LM	3.1 ± 2.0	2.8 ± 2.7	−1.2 (−2.5, −0.005)	0.049	3.4 ± 4.7	−1.5 (−3.5, 0.5)	0.149	2.9 ± 1.7	−0.5 (−1.5, 0.6)	0.358
	LM	2.8 ± 2.2	3.1 ± 2.8			3.7 ± 4.2			3.2 ± 1.6		

## Data Availability

Not applicable.

## Supplementary Materials

The following supporting information can be downloaded at: https://www.mdpi.com/article/10.3390/jcm11164751/s1, Table S1: Type and number of adverse events in the two intervention groups.
