# Supplementary material for: Effects of Fasting and Lifestyle Modification in Patients with Metabolic Syndrome: A Randomized Controlled Trial
Source: J Clin Med. 2022 Aug 14;11(16):4751. doi: 10.3390/jcm11164751 (PMC9410059; doi:10.3390/jcm11164751)
Supplement: Supplementary file 1 [file jcm-11-04751-s001.zip › jcm-1792079-supplementary.pdf]

Supplementary Material

Table S1: Type and number of adverse events in the two intervention groups.

Type of adverse event	Total	F+LM	LM
Tiredness	9	6	3
Decreased physical capacity	2	1	1
Decreased memory	1	1	0
Depressive mood (mild) or sadness	2	2	0
Panic, anxiety	1	0	1
Craniomandibular dysfunction	1	1	0
Headache	5	4	1
Tinnitus, increased	2	1	1
Vertigo	2	2	0
Syncope	1	1	0
Intermittent atrial fibrillation (with known paroxysmal atrial fibrillation)	3	3	0
Bronchitis	4	1	3
Allergic symptoms	2	1	1
Common cold	6	3	3
Fever after vaccination	1	1	0
Gout, episode	1	1	0
Eczema	2	1	1
Thyroid dysfunction	1	1	0
Diabetes, newly diagnosed	2	1	1
Eructation	1	1	0
Nausea	1	1	0
Vomiting	4	4	0
Gastrointestinal symptoms, unspecific	4	4	0
Gastritis	1	1	0
Diarrhea	1	1	0
Incontinence	1	1	0
Urinary retention	1	1	0
Muscular weakness	1	1	0
Back pain	6	4	2
Leg pain	1	1	0
Cramps in the leg	2	2	0
Fracture	3	2	1
Anxiety	1	0	1

Vertigo	1	0	1
Hypertensive emergency	1	0	1
Obstructive sleep apnea	1	0	1
Pain associated with polymyalgia rheumatica	2	0	2
Herpes zoster	1	0	1
Pruritus	1	0	1
Dry eyes	1	0	1
Gingivitis	1	0	1
Tendinitis	2	1	1
Carpal tunnel syndrome	1	0	1
Knee pain	3	0	3
Lightheadedness	7	5	2
Thyroid nodules	1	0	1
Suspected coronary heart disease	1	0	1
Rhinitis	1	0	1
Bladder complaints	1	0	1
Knee effusion	1	0	1
Migraine	1	0	1
Skull / brain trauma after car accident	1	0	1
Syncopation	1	1	0
Baker's cyst	1	1	0
Flu	2	2	0
Cervical spine syndrome with arm paresthesia	1	0	1
Hair loss	1	1	0
Radiculopathy	2	1	1
Prostatitis	1	1	0
Pain in heel spurs	1	0	1
Disc prolaps	1	0	1
Hyperglycemia	1	1	0
Sclerodermia	1	1	0
Osteoarthritis	3	0	3
Osteoarthritis aggravation	1	1	0
Paresthesia after vaccination	1	1	0
Reflux	1	0	1
Total	124	73	51
Abbreviations: F+LM, fasting and lifestyle modification; LM, lifestyle modification.			
